# Transcriptional control of interferon-stimulated genes

**DOI:** 10.1016/j.jbc.2024.107771

**Published:** 2024-09-12

**Authors:** Olga Babadei, Birgit Strobl, Mathias Müller, Thomas Decker

**Affiliations:** 1 Max Perutz Labs, Vienna Biocenter Campus (VBC), Vienna, Austria; 2 University of Vienna, Center for Molecular Biology, Department of Microbiology, Immunobiology and Genetics, Vienna, Austria; 3 Institute of Animal Breeding and Genetics, University of Veterinary Medicine Vienna, Vienna, Austria

**Keywords:** interferon, interferon-stimulated gene, transcription, chromatin

## Abstract

Interferon-induced genes are among the best-studied groups of coregulated genes. Nevertheless, intense research into their regulation, supported by new technologies, is continuing to provide insights into their many layers of transcriptional regulation and to reveal how cellular transcriptomes change with pathogen-induced innate and adaptive immunity. This article gives an overview of recent findings on interferon-induced gene regulation, paying attention to contributions beyond the canonical JAK-STAT pathways.

Since their discovery in the 1950s, interferons (IFNs) attracted attention for a variety of reasons. They are important in innate resistance to viruses, and establishing the antiviral state relies on rapid transcription of IFN-stimulated genes (ISGs). The IFN response has come to represent a paradigm for studying how receptor-derived signals rapidly reach the nucleus to induce transcription. JAK-STAT pathways are the instrument of nuclear signaling by IFN receptors and are widely employed signaling pathways for cytokine and growth factor receptors. The attention on the transcriptional induction of ISG has been reflected in numerous reviews (1, 2, 3, 4, 5, 6, 7). So why yet another one? The simple answer is that IFN signaling continues to be a primary system to tackle new questions or apply new technologies to cytokine-induced transcription. Developments in our understanding of the transcriptional control of ISG are still producing a wealth of insights as material for review. We will be fairly brief in our description of the well-known aspects of transcriptional control and will focus on less covered or very recent developments.

## IFN-stimulated genes

About 10% of protein-coding genes in humans are potentially responsive to IFN (8). However, the number of ISG responding in any given situation depends on several factors. First, the three distinct types (type I, mainly IFNα/β; type II, IFNγ; type III, IFNλ) signal *via* different cell-surface receptors. Nuclear signaling and transcription factor activation by the receptors for type I IFN (IFN-I) and IFN-III are similar, although not identical, and both differ from that of the IFNγ receptor. IFN-III receptors are confined to epithelial cells, hepatocytes (in humans), and subsets of leukocytes, in contrast to the ubiquitous presence of IFN-I and IFNγ receptors (9, 10, 11). Second, type I and to a lesser extent type III IFN are found in subtype families with different signaling characteristics (10, 12). For example, all IFN-I family members assemble a receptor consisting of IFNα receptor (IFNAR)1 and IFNAR2 chains, but their signaling capacity may differ due to differences in the lifetime of the active IFNAR complex (9, 13). This is reflected by quantitative differences in ISG induction between IFN-I subtypes, as shown by a recent scRNA-seq–based study (14). Likewise, low or high concentrations of IFN have differential effects on ISG induction. Third, even when IFN receptor expression is ubiquitous, not all ISG are induced in all cell types, and a majority of ISG show lineage or cell type-restricted responsiveness. We can distinguish between robust and tunable ISG for IFN-I. Robust ISG respond to all IFN subtypes and to low IFN doses, whereas tunable genes show variable responses to IFN-I subtypes, require higher IFN doses, and show cell type–restricted expression patterns (15, 16). A landmark study (17) identified a total of 975 ISG in 11 different cell types, 166 of which formed the ISG core of genes robustly induced by IFN-I in all cell types. This core comprises the genes encoding the well-known antiviral effector proteins (8). We are unaware of any similar studies of core and cell type–dependent IFNγ-induced genes, and the overlap with genes induced by IFN-I and IFN-III in different cells requires further definition.

Not only does signaling by both IFN-I and IFNγ receptors stimulate gene expression but it also represses a large number of genes (termed IrepG or IRG (18, 19)). IFNγ-repressed genes are associated with cell growth or differentiation (19), but we do not know whether this is the only contribution of IrepG/IRG to the biology of the different IFN types. IFNγ signaling also causes repression of genes that inhibits TLR4/LPS-induced macrophage activation (20). Macrophage activation, or M1 polarization, is further enhanced by the inhibitory activity of IFNγ signaling on M2 polarization–associated gene expression (21). Thus, gene activation and repression converge in the generation of activated macrophages as a pivotal immunological function of IFNγ.

ISGs are not controlled by an IFN-dependent toggle that switches between homeostatic inactivity and high, IFN-induced transcriptional activity (22). Instead, homeostasis is characterized by low levels of ISG expression. Taniguchi and Tanaoka (23) described tonic synthesis of small quantities of IFN-I that signal through the IFN-I receptor (IFNAR) complex. The basal expression of different ISG shows variable requirement for the tonic, IFN-derived signal, as reflected by sensitivity to deletion of the IFNAR-associated TYK2 kinase (17). The IFN response of most ISG is controlled by *de novo* Pol II recruitment, but a low degree of preloading and reduced pausing of Pol II may determine an increased speed of mRNA synthesis for some ISG (17, 24). The notion that basal ISG expression warrants an increased response to fully fledged IFN-I signaling upon viral infection is not supported by experiments, which instead demonstrate a lack of correlation between the speed of ISG induction and their dependence on the tonic, TYK2-dependent IFN signal (17). The main importance of homeostatic ISG expression may lie in providing an antiviral ground state as a first line of defense against infection. Tonic IFN signaling is stimulated by the microbiome (25) and signal transducer and activator of transcription (STAT2)/IRF9 complexes contribute to IFN-receptor–independent maintenance of basal ISG expression in mouse macrophages (26). Our recent study of splenic macrophages and T cells showed that tissue context is important for homeostatic ISG expression. Removal of cells from their organ environment causes a dramatic loss of ISG signatures (27). We need further studies to clarify whether tissue context is required for homeostatic IFN-I synthesis and whether additional, cell-contact–dependent signals also contribute.

In addition to the direct transcriptional control of ISG by nuclear signaling, transcriptome changes by IFN may be influenced by, or may require, secondary signals. For example, transcriptional activity induced by IFN produces a memory effect at some ISG, indicated by alterations in histone modification, promoter/enhancer accessibility, and/or 3D chromatin arrangements (28, 29, 30). The memory configuration allows a more rapid and vigorous response of ISG to a second stimulus with IFN and can be stable over many cell generations (29). ISG thus contribute to trained immunity (31). The responsiveness of ISG also varies throughout the cell cycle: recent single cell analysis has shown that a chromatin configuration allowing macrophage M1 polarization by IFNγ is biased toward the G1 phase (32). IFN-I and IFNγ prime different groups of genes for induction by a variety of secondary, proinflammatory stimuli such as TNFα (33). This aspect of IFN signaling is likely to make a strong contribution to inflammatory responses during infection.

## STAT activation by IFN, characteristic attributes of ISG promoters

**Figure 1:**
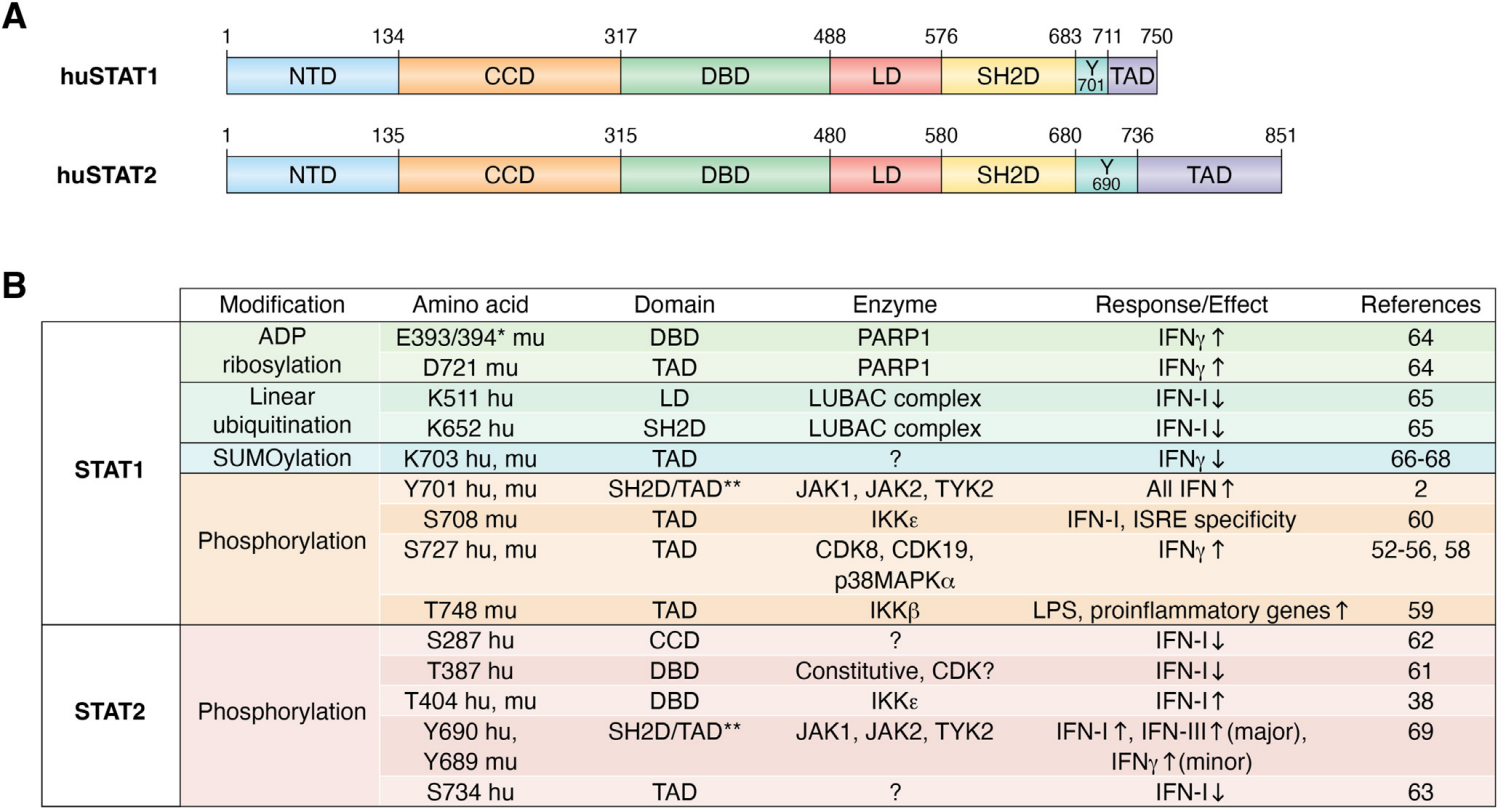
**Posttranslational modifications (PTM) of STAT1 and STAT2 (Uniprot accession numbers:****P42224****huSTAT1; A0A087WSP5 muSTAT1; P52630 huSTAT2; and Q9WVL2 muSTAT2).***A*, domain arrangements of human STAT1 and human STAT2 (2, 69, 106). The position of the tyrosine residue associated with activation is indicated between the SH2D and TAD. The STAT2 DBD shows a large degree of homology to that of STAT1, but it does not contribute to DNA binding of the ISGF3 complex. *B*, list of PTM reported for human (hu) or murine (mu) STAT1 and STAT2. The species designation indicates for which organism the respective PTM was published but is not meant to imply that a similar regulation does not occur in other mammalian species. *Arrows* in the Response/Effect column indicate in which signaling pathway the expression of ISG is either upregulated or downregulated by the respective PTM. ∗Unambiguous assignment not possible; ∗∗ Between SH2 and transactivating domain. CCD, coiled-coil domain; DBD, DNA-binding domain; ISG, IFN-stimulated gene; ISGF3, ISG factor-3; LD, linker domain; NTD, N-terminal domain; SH2D, SH2 domain; STAT, signal transducer and activator of transcription; TAD, transactivating domain.

Canonical signaling by IFN receptors employs receptor-associated Janus kinases (JAKs) to phosphorylate the stimulus-regulated transcription factors (SRTFs) STAT1 and STAT2 on tyrosine, changing their dimerization properties (Fig. 1). Unphosphorylated STAT1 forms antiparallel homodimers, whereas STAT2, but not STAT1, displays a low level of association with IFN regulatory factor (IRF) 9 (26, 34, 35, 36). STAT1 and STAT2 also show a low level of homeostatic preassociation that, at least in part, consists of the unphosphorylated proteins in an antiparallel orientation (26, 37, 38). Tyrosine-phosphorylated STAT1 forms either homodimers oriented in parallel or heterodimers with STAT2 (7). The relative amounts of STAT1 dimers and STAT1-STAT2 heterodimers depend on the IFN type used to stimulate cells. In the case of IFNγ, they also depend on the relative levels of STAT2 because unphosphorylated STAT2 squelches STAT1 and keeps it from forming IFNγ-induced homodimers (39). In general, tyrosine-phosphorylated STAT1 homodimers are most abundant and least transient after IFNγ stimulation, whereas STAT1-STAT2 heterodimers are far more abundant after stimulation with type I or type III IFN. The tyrosine-phosphorylated STAT dimers localize to the nucleus where STAT1 homodimers, also known as gamma-interferon activation factor (40), associate with gamma interferon–activated sites (GASs; (41)). In contrast, STAT1-STAT2 heterodimers form the ISG factor-3 (ISGF3) complex together with IRF9 and associate with IFN-stimulated response elements (42). ISGF3 complex formation from STAT1-STAT2 heterodimers and IRF9 occurs on DNA and not in the cytoplasm, as suggested by most introductions to and graphics of the IFN-I pathway (26, 43).

Mutations of the IFNγ pathway in humans and mice cause susceptibility to intracellular nonviral pathogens, such as mycobacteria, whose elimination requires activated macrophages. In contrast, mutations in the IFN-I and IFN-III pathways largely affect resistance to viruses, suggesting a nonredundant activity in the establishment of the antiviral state (44, 45, 46, 47, 48, 49, 50). The standard description of the canonical pathways attributes the immunological differences between IFNγ *versus* IFN-I and IFN-III to a predominant contribution of genes with GAS promoter elements as opposed to a predominant response of genes with IFN-stimulated response element (ISRE) promoter elements. Genes with both GAS and ISRE elements in their promoters are responsive to all IFN types. This is an important determinant of both different and overlapping attributes of the response to the three types of IFN (51). However, we now understand that considerable mechanistic variability adds complexity to the canonical IFN pathways: there are posttranslational modifications, noncanonical complexes, feed-forward and feedback loops of transcriptional activation, and the establishment of IFN-permissive promoter and enhancer elements during cell differentiation.

## Modulation of STAT1 and STAT2 activity by posttranslational modification

The activities of both STATs 1 and 2 are influenced by phosphorylation of amino acids other than the canonical tyrosine (Fig. 1). In the case of STAT1, the best characterized phosphorylation site is the C-terminal S727, which is phosphorylated both in the IFN pathway and through IFN-independent pathways (52, 53). Mutation of this residue reduces transcription of a subset of IFNγ-induced genes and IFNγ-dependent immune responses (52, 54). During IFN responses, the phosphate is attached by the kinase module of the mediator complex and its constituent cyclin-dependent kinase (CDK)8 or CDK19 enzymes (55, 56). Mediator is a multisubunit complex with regulatory functions in both initiation and elongation of transcription. A recent study suggests a role for the Hippo pathway protein LATS1, phosphorylated by the TYK2 kinase, as a signaling intermediate between the IFN-I receptor complex and CDK8 (57). An alternative scenario posits recruitment of the dual specificity kinase TNK1 to the IFN-I receptor complex and its subsequent phosphorylation of STAT1 at both Y701 and S727 (58).

The STAT1 C terminus is also phosphorylated on T748 (59). Phosphorylation of this residue occurs independently of IFN signaling through IKKβ and promotes the activation of proinflammatory STAT1 target genes at the expense of antiviral and anti-inflammatory genes. Mice harboring a STAT1T748A mutation show increased resistance to LPS. STAT1 is also phosphorylated at S708 which is a substrate of the IKKε kinase. S708 phosphorylation increases association of the ISGF3 complex with ISREs of a subset of antiviral ISG (60).

The association of ISGF3 with ISRE sequences is also modulated by phosphorylation of STAT2 at T387 (61). This phosphorylation is sensitive to CDK inhibitors and decreases promoter binding. IFN-I are weak inducers, but cortisol causes robust T387 phosphorylation, suggesting inhibitory crosstalk between the IFN pathway and glucocorticoid signaling. There have also been reports that a S287 phosphorylation site within the coiled-coil domain has a negative impact on STAT2 activity. When this is mutated to A, a gain-of-ISGF3 function occurs because its association with chromatin is increased. Furthermore, phosphorylation of the C-terminal S734 reduces, and S734A mutation increases, expression of an ISG subset by unknown mechanisms (62, 63). We do not know which kinases phosphorylate these residues although phosphorylation of S734 requires JAK activity. Phosphorylation at T404 has a positive effect on STAT2 activity; it disrupts unphosphorylated STAT1-STAT2 dimers, thus promoting the formation of ISGF3 in IFN-I–stimulated cells (38). Phosphorylation of T404 occurs during viral infection, and the data suggest an important role for IKKε.

In addition to posttranslational modification by phosphates, macrophage STAT1 is modified by ADP-ribose moieties at a glutamic acid residue in the DNA-binding domain and an aspartate residue in the C-terminal transactivation domain. The two modifications increase the activity of STAT1 in IFNγ responses by directing it to the proper genomic binding sites and by increasing both S727 phosphorylation and association with the histone acetylases CBP/p300 (64). Conversely, attachment of linear ubiquitin chains at K652 reduces the interaction of STAT1 with the IFN-I receptor complex, curtailing its tyrosine phosphorylation (65). The modification is removed by the Otulin deubiquitinase as part of the cellular response to IFN-I. Finally, STAT1 is modified by SUMOylation at the C-terminal K703, and this modification is mutually exclusive with phosphorylation at Y701 (66, 67). Mutation of the SUMOylation site causes the formation of a large reservoir of tyrosine-phosphorylated STAT1 in the nucleus and an aggregation to a paracrystalline structure. Consistent with this finding, macrophages from mice without the STAT1 SUMOylation site are hyperresponsive to IFNγ and show an increased sensitivity to the cytotoxic effects of LPS (68).

## Variations of transcription factor complexes containing STAT1 and STAT2

The identification of alternative ISGF3 complexes was sparked by the finding that the kinetics and quantity of ISRE-dependent transcription are altered in IFN-treated cells lacking STAT1 or STAT2, but not both (69, 70, 71). The occurrence of STAT1-IRF9 and, particularly, STAT2-IRF9 complexes was proposed and demonstrated (34, 72). The nature of these complexes in cells is still not entirely clear, although they may result from IRF9 binding to dimerized, tyrosine phosphorylated STAT1 or STAT2 (72). The structure of a phosphorylation-independent STAT2–IRF9 complex has been solved (35), but its structural and functional relationship to the complex containing tyrosine-phosphorylated STAT2 remains to be determined. Importantly, there has been no demonstration that noncanonical complexes lacking STAT1 or STAT2 have a role in ISG transcription in IFN-treated wt cells. Homeostatic expression of ISG is different. In bone marrow–derived murine macrophages, constitutive expression of an ISG subset corresponds with DNA-associated STAT2–IRF9 complexes that switch to ISGF3 upon IFN treatment (26). In splenic macrophages, knockouts of STAT1, STAT2, or IRF9 diverge strongly in their impact on the loss of constitutive ISG expression. While the effects of the STAT2 and IRF9 knockouts showed a high degree of overlap, the STAT1 knockout has markedly different consequences (27), although the three knockouts have similar effects on the ISG core. The data suggest that ISGF3 has a major role in response to tonic IFN-I synthesis and is involved in homeostatic expression of core/robust ISG. In contrast, STAT2–IRF9 complexes are widely used for the basal synthesis of tunable or cell type–specific ISG mRNAs.

Another source of variation in ISGF3 complexes is the existence of the STAT1α or STAT1β isoforms. The STAT1β isoform contains a different, shorter C terminus that lacks a large part of the transactivating domain (TAD), including the S727 phosphorylation site. Although the TAD-less STAT1β has transcriptional activity, mice expressing only the STAT1β isoform show a more drastic effect on IFNγ-induced gene expression and IFNγ-dependent immunity to intracellular bacteria than mice with an S727A mutation (73). IFN-I-and IFN-III-dependent gene expression and immunity are largely unaffected. Bone marrow–derived macrophages expressing only the STAT1α isoform show only subtle changes in IFN-induced transcription. Again, the impact on immune homeostasis in splenic macrophages tells a somewhat different story; the basal expression of a subset of genes with GAS promoter elements requires the presence of both STAT1 isoforms (27). This shows that the two STAT1 isoforms cooperate in the maintenance of homeostatic ISG expression by STAT1 homodimers.

Finally, phosphorylation-independent U-ISGF3 complexes prolong IFN-I signaling and expression of ISG subsets (74) The model posits that the early IFN-I response increases STAT1, STAT2, and IRF9 levels to the point at which they form tyrosine phosphorylation-independent ISGF3 complexes. This mechanism of extended ISG induction has been demonstrated for nonhematopoietic cell lines. It appears to be cell type dependent as studies in both hematopoietic and nonhematopoietic cells have found a direct correlation between JAK activity and ISG expression, also at late stages of the response (24, 71, 75).

**Figure 2:**
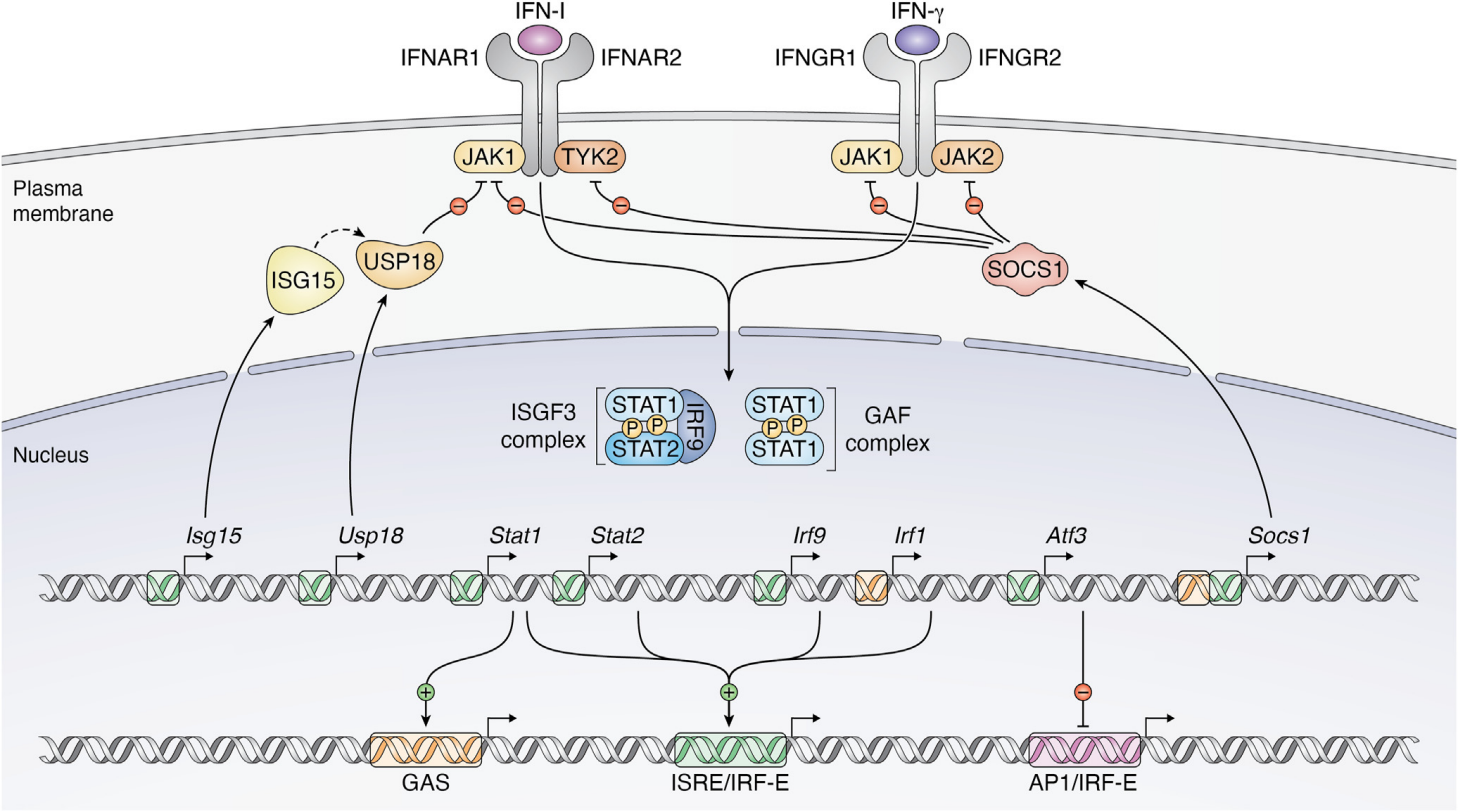
**Feed-forward and feed-back loops of ISG regulation**. Products of ISG such as USP18 or SOCS1 inhibit the IFN response at receptor level, whereas ISG-encoded transcription factors either enhance (STAT1, STAT2, and IRF9; IRF1) further ISG expression, or they act as repressors (ATF3). GAS, gamma IFN-activated site; IFN, interferon; IRF, IFN regulatory factor; GAF, gamma-IFN-activated factor; ISG, IFN-stimulated gene; ISGF3, ISG factor-3; ISRE, IFN-stimulated response element; JAK, Janus kinase; SOCS, suppressors of cytokine signaling; STAT, signal transducer and activator of transcription.

U-ISGF3 signaling is an example of a feed-forward loop of ISG transcription: the ISGF3 subunit genes are themselves induced by IFN (Fig. 2). This mode of amplifying the IFN response is not limited to the formation of U-STAT complexes and responses mediated by STAT2/IRF9 complexes in STAT1 ko cells, as well as by ISGF3 in wt cells, induced synthesis and increased activation of ISGF3 subunits (Fig, 2; 26, 51, 76). The combined use of next generation sequencing–based technologies and mathematical modeling has provided support for the idea that different levels of feed-forward amplification explain the more proinflammatory character of IFN-I- than IFN-III-induced genes (77).

Feedback loops produce inhibitory effects of IFN-induced proteins on IFN receptor signaling, which are important in limiting the proinflammatory effects of IFN (Fig. 2, (78)). Suppressors of cytokine signaling (SOCS) were discovered in the 1990s as a family of widely employed feed-back inhibitors of cytokine signaling (79). Both SOCS1 and SOCS3 are ISG and have an inhibitory effect on IFN receptor signaling. While the KIR domain of SOCS1 acts as a JAK pseudosubstrate and inhibits catalytic activity, SOCS3 targets and inhibits receptor complexes predominantly *via* SH2 domain-mediated association (80, 81). The ISG-encoded ISG15 and USP18 (also called UBP43) also form a feed-back loop to curtail IFN-I receptor activity. The ubiquitin-like ISG15 stabilizes the peptidase USP18. USP18 associates with STAT2 and is thus targeted to the IFNAR2 chain where it displaces JAK1 from the IFNAR and disrupts its activity. The lack of USP18 and ISG15 in humans causes interferonopathies, a group of autoinflammatory conditions characterized by improperly restrained proinflammatory activity of IFN-I (82, 83). Likewise, a STAT2 gain-of-function mutant that fails to traffic USP18 to the IFNAR2 chain results in lethal inflammatory disease (84). A comprehensive proximity labeling study of the type I IFN pathway identified the E3 ubiquitin ligase PJA2 as a negative regulator of IFNAR signaling (85). PJA2 attaches nondegradative ubiquitin moieties to TYK2. This mode of ubiquitination reduces phosphorylation-mediated activation of TYK2 and its subsequent contribution to STAT phosphorylation. Further studies will show whether PJA2 is part of an IFN-induced feedback loop.

## IFN-responsive control regions in their genomic context

Cell differentiation is associated with the marking of transcriptional control elements by histone modifications such as H3K4me1 and by the deposition of pioneer factors such as the Ets family transcription factor (TF) PU.1. In myeloid cells, these events are followed by the binding of more generally available TFs such as C/EBPβ, AP1, and IRF family members (86, 87). The lineage-determining transcription factors (LDTFs) specify enhancers and thus the transcriptional potential of cells within a given lineage. In contrast, the stimulus-regulated transcription factors (SRTFs) of the immune system respond to inflammatory and/or cytokine stimuli, binding to LDTF-containing poised protoenhancers or to nearby enhancers. In the inflammatory responses of myeloid cells, STAT1 binds to poised enhancers containing the LDTFs PU.1, C/EBPβ, IRF4, and ATF3 and JunB (88). In macrophages specifically, basal ISG expression is associated with composite binding sites for the LDTF PU.1 and IRF8 (89, 90). PU.1 and IRF8 associate with ETS-IRF composite elements with the sequence GGAANNGAAA, where GGAA creates specificity for PU.1 and GAAA for IRF8 (91). Deletion of IRF8 reduces constitutive H3K27 acetylation in the IFN control regions of ISG (90), consistent with the presence of this histone modification at transcriptionally active chromatin. In addition to binding at, or in the vicinity of, poised protoenhancers, SRTF, including STATs, can contact and activate latent enhancers; the process requires PU.1 and chromatin remodeling (92).

Histone rearrangement and changes in DNA accessibility are important in transcriptional activation of ISG. In line with this, early studies showed that chromatin remodeling *via* BAF (SWI/SNF family) complexes and the ATPase subunit BRG1 were required prior to, or concomitant with, the transcriptional response of a subset of IFN-I-induced ISG and for the IFNγ-inducible *CIIta* gene (93, 94, 95, 96, 97). Subsequent ATAC-seq studies produced a complex picture of chromatin opening in ISG promoters. Whereas some regulatory sites of ISG promoters are in open chromatin prior to IFN treatment, others become more accessible as a consequence of treatment with IFN-I or IFNγ. ISG control regions that require IFN signaling for increased accessibility can be subdivided into those that remodel nucleosomes in an ISGF3-dependent manner and others that do not require ISGF3 (17, 24, 98). A rather small fraction of ISG require ISGF3 also for homeostatic promoter accessibility.

Beyond the canonical effects of IFN, IFN-induced changes in chromatin accessibility may influence cell fate decisions. A subset of memory B cells emerges during chronic LCMV infection with an ISG signature and an epigenetic landscape shaped by IFNAR signaling (99).

Based on analysis of STAT1/STAT2 binding, a majority of ISG control regions are localized proximal to the transcription start site (26, 100, 101, 102). However, ATACseq-based co-accessibility analysis has shown that remote binding sites of STATs 1 and 2 act as distal enhancers for a subset of ISG (102). IFNs shape transcriptionally active chromatin landscapes both by the removal of repressive and by the deposition of active chromatin marks (11, 90, 103, 104). ChIP-seq has been used to analyze the histone modifications H3K4me1, H3K4me3, H3K9Ac, H3K9me3, H3K27Ac, and H3K27me3 in mouse embryonic stem cells and embryonic fibroblasts: STAT1/2 binding sites are found at active promoters (H3K4me3, H3K27Ac, and H3K9ac), active enhancers (H3K4me1 and H3K27Ac), bivalent chromatin (H3K4me3 and H3K27me3), poised chromatin (H3K4me1 only), and repressed chromatin (H3K9me3 and H3K27me3; (102)).

There is evidence that the histone acetylases CBP/P300 and GCN5 are involved in the activation of ISG promoters (105, 106, 107). The results of co-immunoprecipitation or proximity labeling techniques are consistent with the notion that the ISGF3 complex and STAT1 dimers are directly involved in recruiting chromatin modifiers and remodelers (24, 26, 108). In IFN-treated macrophages, STAT1 is found in proximity to proteins of histone acetylation/chromatin remodeling complexes such as NuA4 (Tip60), SAGA, ATAC, and SRCAP-SWR1 (INO80), the latter being involved in exchange of the variant histone H2AZ (24). In accordance with the nuclear proximity of STATs to the SRCAP-SWR1 (INO80) remodeler, transcriptional control by IFN-I includes the removal of the H2AZ histone variant from ISG subsets in a process requiring the GCN5 histone acetyl transferase, the bromodomain and extraterminal domain (BET) family protein BRD2, and the ISGF3 complex (109). In HeLa cells, co-IP experiments have shown that RUVBL1 and RUVBL2 interact with the transactivating domain of STAT2 (108). These ATPases form scaffolds for many histone modifying/remodeling complexes, including SRCAP-SWR1 (INO80) and NuA4/Tip60 (110).

Somewhat counterintuitively, transcriptional induction of ISG also requires histone deacetylase (HDAC) activity (111, 112). Specifically HDAC 3 is thought to act by increasing the expression of STATs 1 and 2 (113), although HDACs in general may deploy a more global mechanism by releasing the bromodomain-containing BRD4 protein from acetylated histones (114). BRD4 is yet another BET family member required for the transcriptional activation of ISG; it recruits the serine 2 and serine 5 kinases for RNA Pol II phosphorylation (115, 116). Its availability at ISG promoters is increased by HDAC action to promote transcription. The interaction between the BET family members BRD4 and BRD9 in an ISG subset correlates with the recruitment of the noncanonical BAF chromatin remodeling complex (117). BET family proteins thus have critical roles in various aspects of the transcriptional activation of ISG.

## Alterations in the chromatin loop structure at ISG loci

**Figure 3:**
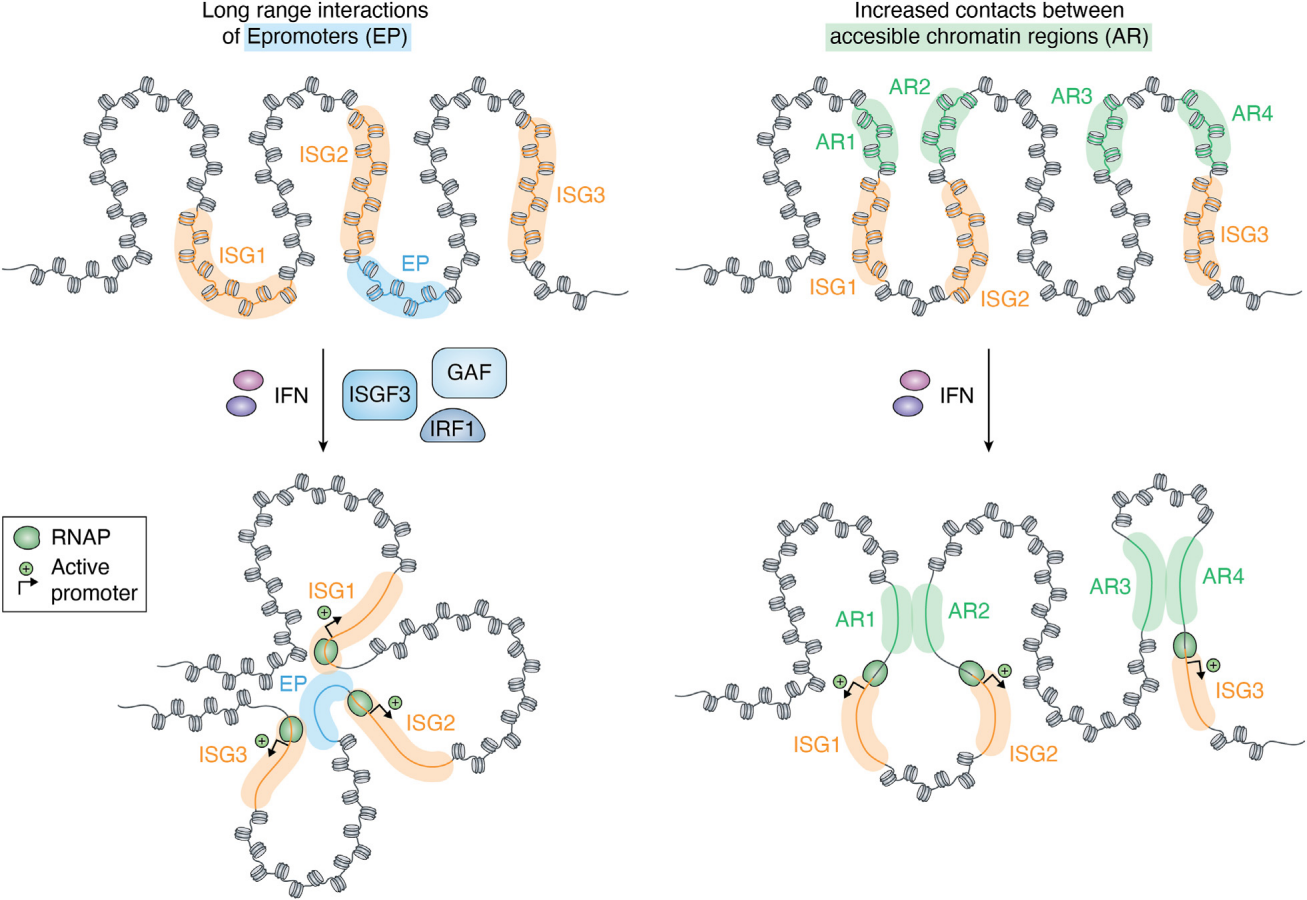
**IFN-induced changes in the chromatin loop structure of clustered ISG, based on data in references 97 and 99**. *Left*: Epromoter sequences act as both IFN-responsive promoters and enhancers to stimulate transcription of adjacent and distant genes in the cluster. The alteration in the loop structure may serve to position IFN response elements in transcription factor-rich nuclear regions and to induce transcription (indicated by *arrows*). *Right*: Consistent with an induced interaction of genomic IFN response elements, both IFN types increase the 3D interaction between nucleosome-free promoter regions (accessible regions, AR) of clustered ISG. GAF, gamma-IFN-activated factor; IFN, interferon; IFN, interferon; IRF, IFN regulatory factor; ISG, IFN-stimulated gene; ISGF3, ISG factor-3.

The 3D structure of chromatin consists of loops of interacting regions that dynamically form, break, and reform (118). The notion that dynamic changes of loop structure contribute to the overall chromatin structure of ISG loci has been the subject of several recent studies, with focus on clustered ISG loci, such as those encoding the guanylate binding proteins (*Gbp*), oligoadenylate synthase genes, or genes for the IFN-induced proteins with tetratricopeptide repeats. HiC studies localized these ISG loci to the A compartment of chromatin also in the uninduced state, indicating that their loop structure is compatible with transcriptional activity. Individual genes of typical ISG clusters have been suggested to localize to the same topologically associated domain (TAD) of a chromosome (29, 98, 100). HiC studies also indicate rapid changes in the ISG loop structure upon treatment with both IFN-I and IFNγ, with a bias toward newly formed loops localizing to intergenic regions with open chromatin, *i.e.*, regions containing control elements (right side of Fig. 3; (98, 104)).

The epromoter concept is compatible with the importance of loop structures of clustered ISG (100). Epromoters act as promoters for one gene and enhancers for a neighboring gene (left side of Fig. 3). Using a combination of STARR-seq-based technologies to identify enhancers and genome editing, the authors showed that typical ISG clusters contain ISGF3-binding sites close to the TSS of one gene and with a regulatory impact on one or more neighboring genes. The data are consistent with the notion of a loop structure that spatially arranges several control regions in an area of increased availability of TFs (100).

A significant body of work has been devoted to the idea that the 3D chromatin structure of ISG shows a transcriptional memory effect. ISG in their memory configuration, *i.e.*, the chromatin state established by a priming treatment with IFN, show an altered landscape of histone modifications and increased deposition of the variant histone H3.3, although this may vary between cell types and ISG (28, 29). The memory state represents chromatin that allows an enhanced association of STATs with their response elements and a more vigorous transcriptional response (30). The 3D configuration of chromatin might have a major role, as suggested by the observation that depletion of cohesin during the priming phase of the IFNγ response increases the memory state of clustered ISG, so cohesin-dependent loop formation may also serve to curb the magnitude of the memory effect on transcription. IFNγ treatment selectively removes cohesins at sites within the TAD containing the *Gbp* cluster (27). However, IFNγ treatment in the priming phase also adds repressive H3K27me3 histone modifications to the *Gbp* locus (104). Transcriptional memory is accompanied by the removal of these marks. A stretch of DNA contacts distant regions in the cluster and curbs an overshooting memory response, emphasizing the importance of long-range chromatin interaction. Further studies are required to determine whether this long-range interaction is related to the effect of cohesin depletion. In addition, an explanation is needed why cohesin depletion interferes with the establishment of a memory effect on nonclustered ISG (29).

## ISG regulation by non-STAT TFs

There are regulators of ISG other than STAT1 dimers and ISGF3. They can be divided into three categories: SRTF required in the context of a STAT-dependent IFN response, such as IRF1 and c-JUN/AP1; SRTF inducing ISG independently of STATs, such as IRF3 and IRF7; and SRTF activated by multiple pathways during an innate response that interact with IFN-activated STATs to generate an adjusted ISG response. Examples of the final category are SRTFs NFκB, AP1 family, and CREB.

## IFN regulatory factors

IRF constitute a family of nine distinct members (119). The genomic binding site, IRF-E, includes a duplicated GAAA repeat found in most ISRE sequences, with the binding of individual IRF dimers modulated by slight variations of the core sequence or the surrounding nucleotides. As a consequence, different IRF-E variants selectively associate with different IRF dimers. Likewise, ISRE variants may constitute high or low affinity IRF-E or select for preferential binding of different family members (101, 120, 121).

## IRF1

The ISGF3 subunit IRF9 and the prototypic family member IRF1 are indispensable for transcriptional responses to IFN. IRF1 was independently identified both through its association with the IRF-E in the IFNβ promoter and as a result of its binding to the ISRE of ISG promoters (122, 123). The *Irf1* gene is an ISG with a GAS in its promoter (124) It is induced by STAT1 homodimers in response to both IFN-I and IFNγ, making IRF1 a second-tier regulator for the transcriptome changes induced by these IFN types. The low amounts of IFNλ receptor expressed in immortalized human hepatocytes result in activation of similarly low levels of tyrosine-phosphorylated STAT1. In consequence, insufficient STAT1 homodimers are produced for robust *Irf1* gene activation and the subsequent transcription of a set of secondary response genes, including proinflammatory chemokines (125). This particular feature of the IFN-III response may explain why it is less proinflammatory than IFN-I. Reduced STAT1/IRF1 feed-forward amplification in the IFN-III response seems consistent with the model for its reduced inflammatory character proposed by Wilder *et al.* (77).

Our recent study in mouse macrophages showed that IFN-I treatment induces a relatively small number of IRF1-dependent genes with delayed induction characteristics. These are not classical antiviral genes (24). IRF1 has long been known to have an essential role in the response of genes characteristic of macrophages activated by IFNγ, such as members of the *Gbp* family or *iNos* that show strongly diminished responsiveness in the absence of IRF1 (90, 107, 126, 127, 128). We and others find that the feed-forward effect of IRF1 synthesis persists much longer in IFNγ-treated than in IFN-I-treated cells (24, 51). This is consistent with the idea that IRF1 has a much larger impact on the delayed structure of the IFNγ-induced transcriptome. The data explain why IFN-I and IFN-γ, although they both mediate rapid synthesis of antiviral and proinflammatory gene products, diverge at later stages, with the IFNγ-induced transcriptome acquiring features of the classically activated M1 macrophage (24). These findings in mouse macrophages are consistent with the effect of human IRF1 deficiency, which results in a mendelian susceptibility to mycobacterial disease, a characteristic of perturbations in IFNγ synthesis or response, including macrophage activation (129). It is important to note that IRF1 synthesis is induced by various inflammatory stimuli originating from pattern recognition receptors or TNF receptors (119, 130). This suggests that IRF1 feeds multiple inflammatory inputs into the transcriptional ISG response, particularly to IFNγ.

## IRF2

IRF2 was originally characterized as an antagonist to IRF1 in the regulation of the IFNβ gene. As IRF1, it is encoded by an ISG (131). Studies of CD8 T cell-dependent skin disease of Irf2−/− mice and of tumor growth resulting from IRF2-dependent CD8 T cell exhaustion concur in showing increased IFN-I–mediated ISG expression in the absence of IRF2, consistent with a repressive role (132, 133). However, IRF2 also acts as an activator of transcription and cooperates with IRF1 in the regulation of genes, including IL12p40, caspase 4, and TLR3 (134, 135, 136, 137, 138). The differential effects of IRF2 on ISG transcription and its relationship to the activity of IRF1 require further investigation.

## IRF3 and IRF7

Unlike IRF1 and IRF2, IRF3 and IRF7 are regulated by phosphorylation and additional posttranslational modifications. They form transcriptionally active dimers when pattern recognition receptors sense microbial infection (139). In contrast to IRF3, IRF7 is expressed at very low levels in resting cells, with the notable exception of plasmacytoid dendritic cells. Both family members are essential regulators of IFN-I and IFN-III genes. IRF3 rapidly induces transcription of the *Ifnb* gene. In contrast, IRF7, which is encoded by an ISG, is synthesized in response to early IFNβ and constitutes a feed-forward loop for the synthesis of the other IFN-I (139, 140).

Active forms of both IRF3 and IRF7 stimulate expression of a subset of ISG independently of IFN synthesis (141, 142). It is therefore likely, although not conclusively shown, that the constitutively expressed IRF3 acts as a rapid, IFN-independent stimulator of ISG transcription. A contribution of IRF7 to ISG transcriptional control is suggested by our finding that IRF7 is required for the maintenance of IFNγ-induced *Gbp2* expression (143). However, we still lack evidence for a more general input of IRF7 to ISG transcription. Many tissues of bats contain high levels of IRF1, IRF3, and IRF7 and use these regulators for the IFN-independent induction of specific subsets of ISG (144). The direct control of ISG expression by IRFs may contribute to the ability of bats to coexist with a variety of viruses, enabling them to serve as reservoirs of infection.

## IRF8 and its interaction with IRF1

IRF8, originally called interferon consensus sequence binding protein, was identified as an IFNγ-induced protein that associates with ISRE sequences (145, 146, 147). It was subsequently shown to confer IFNγ inducibility to ISG in myeloid cells and to cooperate with both PU.1 and IRF1 in this process (148, 149). IRF8 binds to enhancers marked by PU.1 in resting cells and acquires additional binding sites such as the AP1-IRF1 composite element through activities of inflammation-induced SRTF (89). Further comprehensive analysis of IRF8 and IRF1 cistromes and regulomes in macrophages has convincingly shown that the two IRF family members bind promoters together and cooperate in the induction of IFNγ-induced genes, although they also act independently of each other (90). Unlike IRF1, IRF8 is mostly prebound to its target sites, with little change observed after IFNγ treatment. Consistent with this, marks of active chromatin, particularly H3K27Ac, require IRF8 in resting cells and IRF1 after stimulation with IFNγ. IRF8 is expressed predominantly by myeloid and lymphoid cells and confers cell type specificity to the IFNγ response in these lineages, possibly by interacting with, or alternative to, IRF4 (88). IRF8 has also been linked to selective ISG inhibition (150, 151). The molecular context that renders IRF8 repressive requires further investigation.

## SRTFs regulated by infection and inflammation

Innate immune responses to pathogen-derived signals establish a complex network of SRTF that cooperate or antagonize one another (152, 153). Cooperative signals may increase both the antimicrobial and the proinflammatory character of the response to IFN. Reportedly, the latter situation is a major contributing factor to severe Covid (154).

## AP1 family and CREB

Stress-induced MAPK pathways influence STAT1 activity both through phosphorylation of the C-terminal S727 (53) and independently of STAT1 phosphorylation (3, 155, 156). The STAT1 phosphorylation-independent scenario suggests cooperation with SRTF activated through MAPK pathways. In agreement with this notion, AP1/c-JUN has a role in the selective induction of several ISG by IFNγ (157). This finding is consistent with the identification of composite AP1-IRF–binding sites (89, 158, 159). AP1/STAT coregulation of ISG also emerged from our recent study of global transcriptional effects of stress-induced JNK and p38MAPK pathways. A large, IFN type-specific contingent of ISG showed stress pathway–enhanced expression after both IFN-I and IFNγ stimulation (103). The promoters of these genes contain binding sites for the TFs AP1/c-JUN and CREB or both. Deletion of the two factors showed that CREB has a major effect and AP1/c-JUN a minor effect on the enhancement of ISG expression. Surprisingly, the transcriptional activity of CREB does not require the canonical phosphorylation at S133, which allows association with CBP/P300. Stress pathway activation alone does not activate transcription of the stress-enhanced ISG, suggesting that CREB and AP1 must cooperate with STATs.

The AP1 family member ATF3 acts as an LDTF at a subset of IFN control regions (see above, Fig. 2). It has been reported to dissociate transiently from ISG promoters in LPS-treated cells (88) suggesting it may either be degraded or actively removed as part of transcriptional ISG activation. The *Atf3* gene is induced by IFN-I (24, 160), the protein shows proximity in BioID studies to both STAT1 and IRF1 in the nucleus of IFN-treated macrophages (24) and negatively regulates both IFNβ synthesis and an ISG subset (160). The data can be reconciled by proposing that ATF3 curbs basal expression and interacts with ISG promoters at a delayed stage of the IFN response, acting again as a transcriptional repressor and feed-back inhibitor (Fig. 2).

## NFκB

NFκB is activated in response to virtually all perturbations of immune homeostasis. The TF is one of the essential components of the innate immune system’s proinflammatory SRTF network (161). The first gene that IFN signaling was shown to regulate together with NFκB was the CXCL10 chemokine gene, then known as IP10 (162). Follow-up studies identified many chemokine genes as targets of both NFκB and IFN-activated STATs (163). Bioinformatic analysis (164) and ChIP-seq studies (165) showed a frequent interaction of STAT and NFκB pathways at the promoters of genes encoding both proinflammatory mediators and antimicrobial effector proteins. When deciphering mechanisms of NFκB-ISGF3 cooperativity, we found that NFκB deposition at promoters induces histone marks of transcriptionally active regions and recruits the basal TF TFIIH as well as the kinase module of the mediator complex. ISGF3 subsequently recruits the core mediator complex and RNA Pol II (165, 166). NFκB and STATs thus interact in the assembly of essential components of transcriptional activation and in the configuration of transcriptionally active ISG promoters.

## Conclusion

More than 30 years of JAK-STAT research have produced a wealth of information on how IFN uses the pathway to produce antimicrobial immunity and how it induces the genes for its effectors. However, a closer look reveals increased complexity and spawns a plethora of questions. Future research on ISG control will have to accommodate an ever-increasing number of regulators because the IFN response is more often than not embedded into a complex innate immune response. Nonprotein regulators such as long noncoding RNAs are emerging as additional players, although we still have little insight into their impact and mode of action (104, 167). The spatial organization of ISG chromatin, particularly when it contains ISG clusters, are only beginning to emerge. Likewise, the reasons for the cell type-restricted response of ISGs noted in several studies require investigation. Finally, the activities of particularly STAT2 and IRF9 beyond controlling ISG (27) present an attractive area for future research.

## Conflict of interest

The authors declare no conflict of interest with the contents of this article.
